# Thermal Stability of Fluorescent Chitosan Modified with Heterocyclic Aromatic Dyes

**DOI:** 10.3390/ma15103667

**Published:** 2022-05-20

**Authors:** Dagmara Bajer, Halina Kaczmarek

**Affiliations:** Faculty of Chemistry, Nicolaus Copernicus University in Toruń, Gagarina 7, 87-100 Toruń, Poland

**Keywords:** fluorescent chitosan derivatives, heterocyclic aromatic substituents, thermogravimetric analysis, evolved gas analysis

## Abstract

Fluorescent biopolymer derivatives are increasingly used in biology and medicine, but their resistance to heat and UV radiation, which are sterilizing agents, is relatively unknown. In this work, chitosan (CS) modified by three different heterocyclic aromatic dyes based on benzimidazole, benzothiazole, and benzoxazole (assigned as IBm, BTh, and BOx) has been studied. The thermal properties of these CS derivatives have been determined using the Thermogravimetric Analysis coupled with the Fourier Transform Infrared spectroscopy of volatile degradation products. The influence of UV radiation on the thermal resistance of modified, fluorescent chitosan samples was also investigated. Based on the temperature onset as well as the decomposition temperatures at a maximal rate, IBm was found to be more thermally stable than BOx and BTh. However, this dye gave off the most volatile products (mainly water, ammonia, carbon oxides, and carbonyl/ether compounds). The substitution of dyes for chitosan changes its thermal stability slightly. Characteristic decomposition temperatures in modified CS vary by a few degrees (<10 °C) from the virgin sample. Considering the temperatures of the main decomposition stage, CS-BOx turned out to be the most stable. The UV irradiation of chitosan derivatives leads to minor changes in the thermal parameters and a decrease in the number of volatile degradation products. It was concluded that the obtained CS derivatives are characterized by good resistance to heat and UV irradiation, which extends the possibilities of using these innovative materials.

## 1. Introduction

Fluorescent compounds are now widely used in various industrial branches and fields of modern medicine, such as imaging materials, photosensitizers, light-emitting diodes, and biological markers, allowing for the detection of protein, nucleic acids, living cells, or damaged tissues [1,2,3,4,5]. Particularly noteworthy is the use of fluorescent dyes in medical diagnostics, e.g., the early detection of cancer (e.g., neurologic tumors, spinal glioma, thyroid, and parathyroid tumors; breast, colon, and skin cancers) or HIV [6,7,8]. Among those compounds are methylene blue, fluorescein, and indocyanine green. Another group of fluorescent moieties used in anticancer Photodynamic Therapy (PDT) is BODIPY—dyes based on boron difluoride and dipyrromethene groups with various substituents.

As reported by the latest works, fluorescence labeling can also be successfully applied in biological studies and in the detection of the COVID-19 coronavirus [9,10]. Such wide applications are the reason for the intensive research on fluorescent compounds in recent years, contributing to significant progress in modern technologies utilizing light emission phenomena. For practical applications, the specific properties of such compounds are required, namely, a high absorption coefficient, a high fluorescence quantum yield, sensitivity, stability, and a resistance to chemical and environmental factors (including atmospheric oxygen, solar radiation, temperature, and humidity, as well as pollution in the form of exhaust fumes and industrial dust) [1,11].

Biological activity (e.g., antimicrobial, antiviral, or anti-inflammatory properties) and enhanced thermal resistance are required in some cases. Moreover, water solubility, a rare feature in organic compounds, is also substantial for biomedical applications [1,12,13].

The desired properties of fluorescent substances can be obtained in the process of the modification of known organic dyes [14,15,16]. One way to modify low molecular weight organic dyes is through their chemical or physical binding with macromolecules. Recently, several articles on fluorescent polymers [17,18,19], including biopolymers [20,21], have been published, but there are still few studies on the behavior of these innovative materials at elevated temperatures [20,22].

A thermogravimetric analysis of chitin and chitosan grafted by 3-hexylthiophene (3HT) and fluorene (F) was presented in a work by Hai, T.A.P. [20]. It turned out that both functionalized polysaccharides were fluorescent, but their thermal stability decreased compared to the neat biopolymers. However, a physical mixture of chitin or chitosan with conjugated polymers based on 3HT or F exhibited improved thermal resistance. Pan, X. [22], who synthesized novel fluorescent carbazolyl-pyridinyl alternating copolymers, found their thermal stability with an onset of degradation at 370–400 °C and high glass transition (T_g_ = 120–150 °C). These two examples show that it is difficult to predict the heat resistance of polymers modified to achieve emission properties. Therefore, the lack of thermal characteristics of fluorescent polymers, relevant from the point of view of practical applications, is an information gap.

It should be pointed out that the Thermal Analysis (TA) of polymeric materials provides information not only about their heat resistance and uses in various conditions but also about their chemical structure, phase transformations (melting point, glass transition), polymorphic forms, relaxation processes, composition and purity of compounds, constituent compatibility, degradation products, and kinetics. It is also a valuable tool for the quick identification of adsorbed volatile substances, e.g., moisture content (also the determination of crystallization water), residual solvents, unreacted monomers, and modifiers [23,24,25]. TA, which can be conducted in various atmospheres (oxidizing or inert), covers a whole range of techniques, ranging from classical Thermogravimetry (TG) or Differential Scanning Calorimetry (DSC) to more complex techniques, e.g., TG-DTG (Derivative Thermogravimetry), TG-DTA (Differential Thermal Analysis), Thermomechanical Analysis (TMA), and Dynamic Mechanical Analysis (DMA) [26,27].

Moreover, thermogravimetry can be coupled with instruments, allowing for the monitoring of the evolved gas, such as MS (Mass Spectrometry), FTIR (Fourier Transform Infrared Spectroscopy), or GC-MS (Gas Chromatography-MS). The use of online combined (called also hyphenated) techniques, which exploit the advantages of the individual components, provides quick and complementary results about the material under study [23]. These methods allow you to record simultaneously, among other things, weight loss, energetic effects (Exo and Endo), heat capacity, the characteristic temperatures (i.e., the onset or the maximum rate) of physical processes and chemical reactions, or the type of gaseous substances released as a function of temperature. These multi-parameter results are obtained during one measurement in the same conditions for a small sample (a few mg) [28].

Hyphenated thermal analysis techniques (double or even triple) are currently widely used to study the thermal processes in polymers [29] and polymeric nanocomposites [30,31] and, increasingly, for the study of waste pyrolysis [32] and microplastics polluting the environment [33,34].

Our last work described the photochemical properties of chitosan modified with heterocyclic aromatic dyes based on benzimidazole, benzoxazole, and benzothiazole [35]. It was found that even a small degree of the replacement of functional groups in chitosan with fluorophores (not exceeding 3%) contributes to the formation of intensive fluorescence when excited at a wavelength of 425 nm. Moreover, new fluorescent chitosan derivatives were tested for their photochemical stability and biological properties. The effect of the high-energy UV-C radiation with a wavelength of 254 nm was studied for both solid samples and solutions using FTIR and UV-Vis spectroscopy. It has been proven that photochemical reactions (mainly photobleaching) occur with greater efficiency in solutions than in the thin solid films of modified chitosan [35]. The use of chitosan as a matrix for fluorescent compounds has many advantages, the most important of which is the biodegradability of the system and an increase in the photochemical stability of the substituted dyes. Furthermore, these modified chitosan specimens are characterized by biocompatibility, biological inertness, and biocidal properties.

The main goal of this work was to present the results of the thermogravimetric analysis coupled with the FTIR analysis of evolved gas for three fluorescent chitosan (CS) derivatives containing substituted *trans*-2-[2-(4-formylphenyl)ethenyl]benzimi-dazole, BIm, *p-*[*trans*-2-(benzoxazol-2-yl)ethenyl]benzaldehyde, BOx, and *p*-[*trans*-2-(benzthiazol-2-yl)ethenyl]benzaldehyde, BTh. The thermal stability of the dyes (BIm, Box, BTh) used to modify chitosan was also investigated. TA-FTIR was applied to illustrate the chemical changes in the structure of the modified fluorescent chitosan under the influence of UV irradiation.

This work is a continuation of the characterization of the same N-substituted chitosan derivatives presented in the previous work [35]. Information about the thermal properties of these new polysaccharide materials is essential from a practical point of view—it allows us to estimate the possibility of their use at elevated temperatures. At the same time, TGA tests enable the detection of structural changes in the case of the prior exposure of samples to UV radiation, often used as a sterilizing agent.

## 2. Materials and Methods

### 2.1. Materials

**Figure 1:**
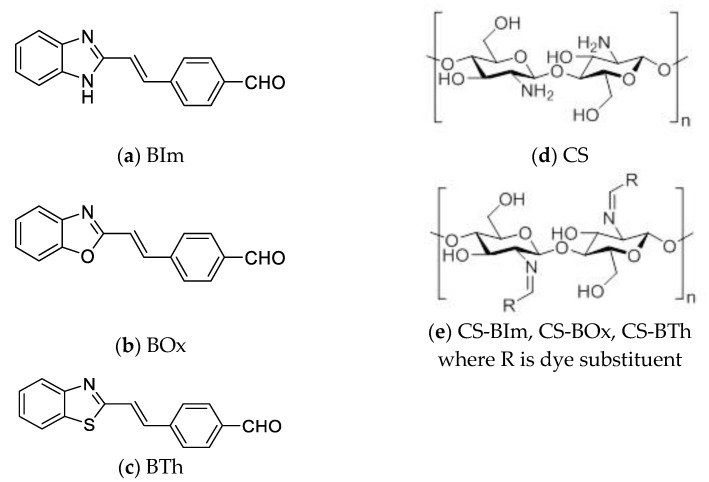
Structure of studied compounds—dye: BIm (**a**), BOx (**b**), and BTh (**c**); initial chitosan as a reference (**d**) and fluorescent chitosan CS-BIm, CS-BOx, and CS-BTh (**e**).

Chitosan (CS), a copolymer of β-(1→4)-linked D-glucosamine with *N*-acetyl-D-glucosamine, and substrates for the synthesis of the dyes (2-methylbenzimidazole, 2-methylbenzoxazole, 2-methylbenzothiazole, and terephthalaldehyde) have been purchased from Sigma-Aldrich (St. Louis, MI, USA). Other reagents and solvents (acetic anhydride, acetic acid, acetone, and methanol) were supplied by Avantor™ Performance Materials, Gliwice, Poland. The average molecular weight and deacetylation degree of the initial chitosan were 50,000 g/mol and 87%, respectively. The synthesis of the dyes (BIm, BOx, BTh) was performed according to the procedure described in work [36]. These compounds were substituted into the biopolymer chains using the reaction of the aldehyde groups of the dyes with the amino groups in CS. The chemical structures and abbreviations of the studied compounds are shown in Figure 1.

The modified chitosans contained a relatively low amount of dye substituents, i.e., 2.9%, 2.7%, and 2.4% in CS-BIm, CS-BOx, and CS-BTh, respectively. The thin films (Figure S1) were made from the obtained chitosan derivatives by pouring dilute acetic acid solutions onto leveled 8 cm Petri dishes and evaporating the solvent at room temperature. A detailed description of this modification and the methodology of the sample preparation for testing were included in the earlier publication [35].

Half of the films of the chitosan derivatives were subjected to UV irradiation in room conditions for 8 h using a low-pressure mercury-vapor lamp TUV-30 W produced by Philips, Holland. The lamp emitted radiation with a wavelength of 254 nm, and the dose obtained by the samples was 28.8 J/cm^2^. Samples of the same thickness (approx. 10 μm) were selected for irradiation.

### 2.2. Thermal Analysis

The thermogravimetric analysis (TGA) of all samples (non-irradiated and UV-irradiated) was performed on the Jupiter STA 449 F5 thermoanalyzer by Netzsch coupled with the FT-IR Vertex 70V spectrometer by Bruker Optik in the following conditions: a nitrogen atmosphere, a temperature range of 20–600 °C, and a heating rate of 10°/min. The weight of the samples was 6–10 mg. The simultaneous measurement of TG, DTG, and DSC allowed for the determination of the following parameters: decomposition onset temperature (T_0_), temperature (T_max_) at the maximum process rate (V_max_), weight loss (∆m) for individual stages, and heat effects (ΔH) accompanying the decomposition. The mixture of volatile products, released at temperatures ranging from room temperature to 600 °C, was analyzed based on the infrared spectra recorded in the range of 400–4000 cm^−1^. The evolved gases were transported to the gas cell (with ZnSe spectrophotometric windows) in an FTIR spectrophotometer. The connections between the thermoanalyzer and spectrophotometer were heated to 200 °C to prevent condensation. The carrier gas shows no infrared absorption.

## 3. Results and Discussion

The thermogravimetric analysis of specially synthesized heterocyclic compounds and chitosan modified with those dyes, as well as the influence of UV radiation on the thermal stability of the samples, were tested under dynamic conditions. The investigations included the in situ detection of gaseous products released during heating (using FTIR spectroscopy). Combining these two techniques allows for an understanding of the thermal decomposition mechanism of new fluorescent chitosan derivatives, which have not been the subject of research so far.

### 3.1. Thermal Stability of Chitosan Modifiers: Low-Molecular-Weight Heterocyclic Compounds—BIm, Box, and BTh

**Figure 2:**
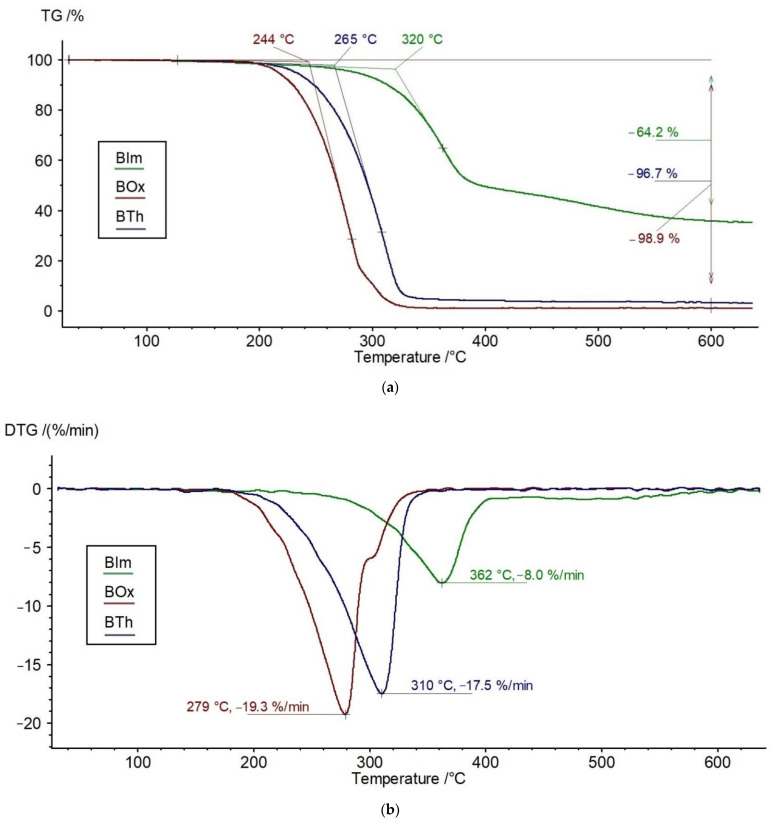

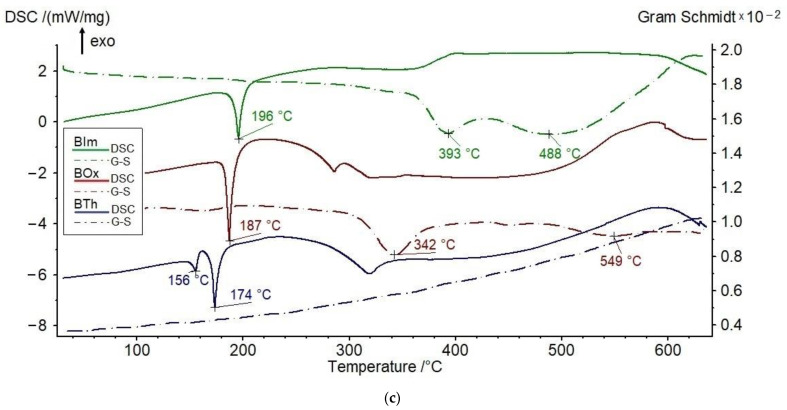
TG (**a**), DTG (**b**), DSC, and Gram–Schmidt graphs (**c**) of BIm, Box, and BTh compounds.

The TGA shows that the course of the thermal decomposition of the three tested heterocyclic aromatic dyes differs significantly, which of course results from the difference in the chemical structure of these compounds (Figure 2a–c). In any case, no evaporation of moisture is observed (no weight loss around 100 °C). Two of these compounds (BIm and BTh) are destroyed in one step. Only BOx shows two visible on the DTG curve stages: the main with a maximum at 279 °C (with dominant mass loss) and the overlapping minor step at about 300 °C (Figure 2b).

Taking into account the temperatures characterizing the decay onset, the BOx sample has the lowest thermal resistance (T_o_ is 244 °C). In contrast, the most durable is BIm, which begins to decompose only around 320 °C (Table 1). Additionally, the maximum rate of the BOx decomposition is the lowest compared to the other two.

The weight loss of BIm at 600 °C is approx. 64%, while BOx and BTh decompose almost completely (∆m > 96%). These variations can be explained by the different stabilities of the heterocyclic ring, in which the weakest point is a single C-heteroatom linkage. It should be emphasized that the studied molecules are stabilized by resonance due to the presence of π electrons from aromatic rings, central vinyl bonds, as well as non-bonding electrons at the O, N, and S atoms. The most significant thermal stability in the case of BIm can be explained by the presence of hydrogens at the N atoms in the rings, which can form hydrogen bonds with the N or O atoms from neighboring molecules. Therefore, it can be assumed that these hydrogen bonds between the heterocyclic rings contribute to the increased thermal resistance of BIm. There is no such possibility in BOx and BTh, although, of course, in all of the studied heterocycles, there are aldehyde groups that participate in the formation of hydrogen bonds between the end groups.

The relatively high residue of the BIm sample at 600 °C, compared to the other two, indicates the formation of stable carbonaceous cross-linked structures containing aromatic rings. In the least stable sample BOx, due to the presence of an oxygen heteroatom, primary degradation products with oxidizing properties can be formed, which contributes to the greater degradation of the residue. As can be seen from the results, sulfur has a similar effect.

To establish an order of the stability of the tested compounds, the values of the onset of the decomposition temperature and the temperature at the maximum decomposition rate from TGA (Table 1) were used. It is as follows:

BIm > BTh > BOx

It can be concluded that the thermostability of these three heterocyclic compounds follows a similar trend as their photostability, tested in diluted solutions using an identical source of UV radiation [30].

The shape of the DSC curves also differs depending on the type of sample (Figure 2c). The studied heterocycles show endothermic peaks on the DSC corresponding to the melting point. Only BTh exhibited two separated melting temperatures (157 and 174 °C), indicating the coexistence of two different crystalline forms. The endotherms at higher temperatures correspond to the main decomposition of the samples.

The Gram–Schmidt (G–S) curve of BIm exhibits two extrema at 393 °C and 488 °C (very broad), pointing to the most intense release of volatile decomposition products in these temperatures (Figure 2c). Similarly, in the BOx sample, two main extrema appear at 342 and 549 °C. Only the BTh sample shows a continuous, almost monotonic increase in the G–S curve with temperature, without any peaks. It should be added that the extremum points at the G–S curves do not correspond exactly to the T_max_ read from DTG. This is understandable because the samples do not just decompose into volatile products.

**Figure 3:**
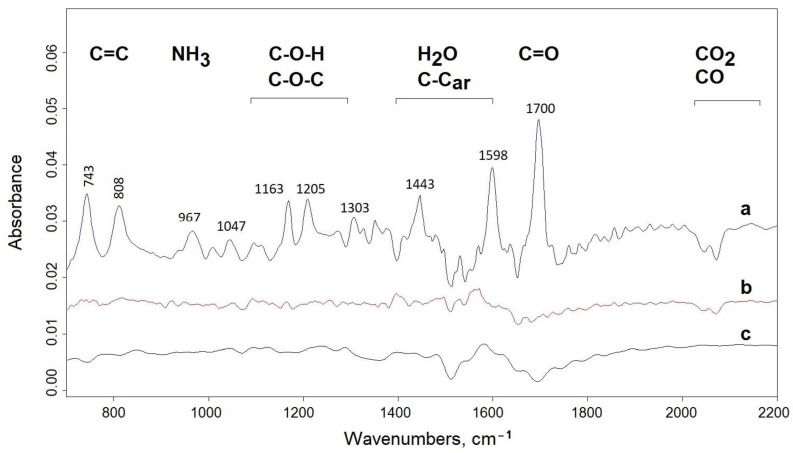
FTIR spectra of evolved gases from BIm (**a**), BOx (**b**), and BTh (**c**) at 500 °C. At the top, the main bands are assigned to the respective groups.

The accurate identification of gaseous products is difficult due to their variety and small quantities. The simultaneous FTIR analysis shows that the greatest number of volatile products was separated from the BIm sample. In addition to the moisture (3000–3600 cm^−1^), the other low intensive bands were observed at 1695, 1596, 1445, 1207, 1168, 966, 809, and 740 cm^−1^, which indicates the evolution of the fragmentation products containing carbonyl, ether, and vinyl groups (Figure 3a) [37]. Nitrogen-containing compounds cannot be excluded, because the absorption of amine/amide (1655 amide I, 1523 amide II, and 1580 N-H) overlaps with the carbonyl region. The weak band at 966 cm^−1^ can be assigned to ammonia.

In the remaining two compounds—BOx and BTh—the number of volatile products registered in infrared is negligible and on the noise limit (Figure 3b,c), suggesting that the released products condense.

It can therefore be concluded that the primary reactions of the thermal decomposition of BIm with the breaking of covalent bonds and the evolution of gaseous products lead to the formation of free radicals, which then recombine into more stable products (not decomposing even at 600 °C, where the carbon residue is above 35%).

### 3.2. Thermal Stability of Chitosan Derivatives—CS-BIm, CS-BOx, CS-BTh

**Figure 4:**
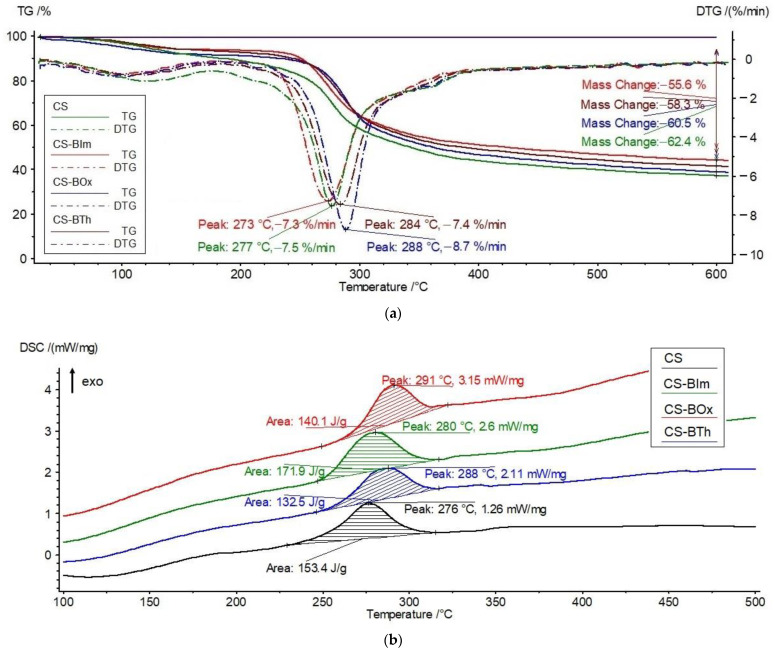
TG/DTG (**a**) and DSC (**b**) curves of CS, CS-BIm, CS-BOx, and CS-BTh (unirradiated).

The shape of the TG, DTG, and DSC curves of the samples heated at a constant rate of 10 °C/min to 600 °C changes slightly (Figure 4), indicating a minor influence of chitosan modification on the course of its thermal degradation.

Chitosan is a stable biopolymer up to about 250 °C. However, in the initial heating stage (in the range of 80–150 °C), a weight loss of approximately 8%, caused by the release of adsorbed water, is observed (Table 2). The similar thermal stability of chitosan has been reported in the literature [38,39]. The data indicate that the water is somewhat more strongly bound in the two *N*-substituted derivatives, CS-BIm and CS-BTh, than in the original chitosan.

The main decomposition of CS starts at 254 °C and proceeds at a maximum rate of 277 °C. It is accompanied by a weight loss of about 55%. Thermal decomposition is an exothermic transformation, as shown by the DSC curves. This exothermic effect can be connected with the simultaneous crosslinking of chitosan, as evidenced by a large residual mass not decomposed at 600 °C. As was reported before by Moussou et al., the apparent activation energy of CS thermal decomposition, determined by the Ozawa–Flynn–Wall method, is about 146 kJ/mol [40].

The shape of the TG, DTG, and DSC curves of CS-BIm, CS-BOx, and CS-BTh is similar to the unmodified chitosan curves, but shifts in the main determined thermal parameters are observed. An increase in T_o_, T_max_, and T_exo_ at the DSC curve in the main decomposition stage (step II) indicates an improvement in the thermal resistance of CS-BOx and CS-BTh compared to CS. In the light of these data, CS-BIm appears to be the least thermally resistant material, although the differences in the determined parameters are low. This can be explained by the lower thermal crosslinking degree, which the smallest amount of carbonaceous residue (55.6%) indicates. This sample exhibits the highest value of decomposition heat (172 J/g).

Considering the total weight loss in the modified chitosan samples, several percent lower than in the pristine CS, it can be concluded that the aromatic heterocyclic substituents slightly hamper the formation of thermally stable carbonaceous residue. Since, in the initial stages of the thermal destruction of polysaccharides, the side substituents are detached from the macrochains with the simultaneous release of gaseous products [41,42], CS loses its functional properties. Obviously, in the case of *N*-substituted chitosan derivatives, the abstraction or destruction of fluorophores (dye substituents) leads to a loss in emission properties. It can be assumed that the carbon residue consists mainly of crosslinked and condensed aromatic structures resulting from formed covalent bonds between the macrochains and the graphitization process, similarly as in unmodified CS [41,42]. This was confirmed by spectroscopic studies carried out systematically while heating the samples. The FTIR spectra showed the disappearance of functional groups (OH, NH) and a decrease in the degree of deacetylation, followed by chain breakage and the opening of glucoside rings. Finally, only the bands characteristic of C-H bonds in the aliphatic and aromatic compounds (corresponding to both stretching and deformation vibrations) were detected in the spectra of thermally degraded CS.

The slight variations in the determined thermal parameters of dye-modified chitosans are due to the low degree of *N*-substitution by heterocycles: 2.4–2.9% [35]. The lack of endothermic melting peaks of BIm, BOx, and BTh on the DSC curves of CS-BIm, CS-BOx, and CS-BTh additionally confirms that these modifying dyes are covalently linked to CS.

Summarizing this part of the research, the following sequence of thermostability can be proposed for the unirradiated samples:

CS-BOx > CS-BTh > CS > CS-BIm

This order was established from the temperature values at a maximum rate (T_max_) for the main (II) decomposition stage, listed in Table 2.

The explanation of the observed effects is not trivial. The reverse trend can be seen: the least stable dye (BOx), when chemically incorporated into CS macromolecules, improves its stability (CS-BOx). Considering the free radical mechanism of thermal decomposition, it can be assumed that benzoxazole radicals generated from the least stable substituent (BOx) quickly recombine with CS macroradicals (thus, finishing the degradation chain reaction), delaying the overall decomposition process.

On the contrary, BIm has the opposite effect, i.e., being the most thermostable dye itself, it generates radicals initiating chitosan degradation faster. Probably, the benzimidazole radicals are responsible for this effect due to the easier reaction with CS.

### 3.3. Effect of UV-Irradiation on the Thermal Stability of Chitosan Derivatives

**Figure 5:**
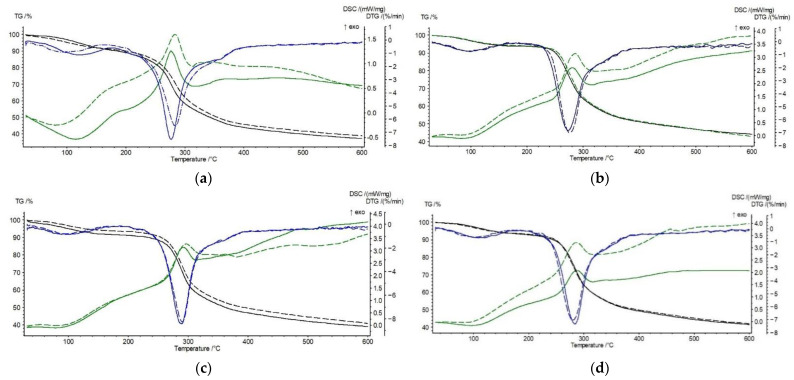
Comparison of TG, DTG, and DSC curves for unirradiated (solid lines) and 8 h UV-irradiated (dashed lines) samples: CS (**a**) CS-BIm (**b**), CS-BOx (**c**), and CS-BTh (**d**).

The thermal analysis also allows for the observation of changes in the modified chitosan samples under UV radiation (Table 3, Figure 5). As can be seen from the presented data, the thermal stability of the modified chitosan previously exposed to UV changes slightly.

The water content in the CS-derivatives is lower by about 3% than in CS. To compare the thermal stability of the exposed CS-BIm, CS-BOx, and CS-BTh, we consider the main (II) stage of decomposition.

Among the UV-irradiated samples, the CS-BOx shows the most significant stability (as evidenced by the value of T_max_ and T_exo_), which may indicate a particular share of oxygen-containing decomposition products in the creation of thermostable structures in this biopolymer (e.g., crosslinking). However, the maximum decomposition rate of this sample is the highest (V_max_ = 8.2%/min). Similarly, the same sample not exposed to UV radiation was characterized by the highest decomposition rate (Table 2). It should be recalled that the starting BOx showed the lowest thermostability among the three tested heterocyclic compounds. This suggests that its degradation products may contribute to the thermal stabilization of CS. The abstraction of dye side groups during pyrolysis followed by their decay to free radicals may lead to subsequent recombination with the CS macroradicals formed in the earlier stages of thermal degradation.

The exposure to UV radiation only resulted in a 2–6.5 °C increase in T_max_ in the CS alone, CS-BIm, and CS-Box samples, while in the CS-BTh sample, the T_max_ decreases by two degrees.

Additionally, the loss of mass during the thermal decomposition of the irradiated samples differs slightly from that in the non-irradiated samples. It can therefore be concluded that the samples of modified CS are resistant to heat and, like chitosan itself, decompose only above 250 °C.

**Figure 6:**
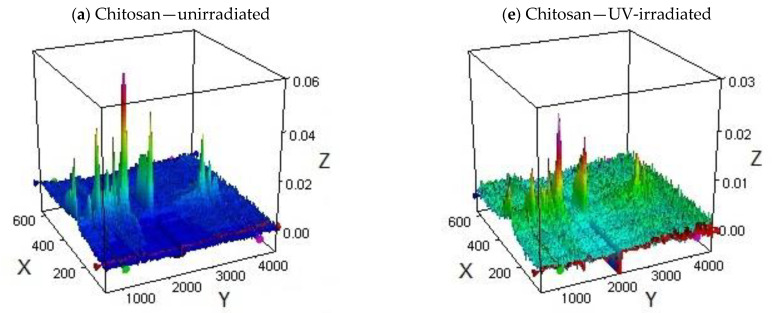

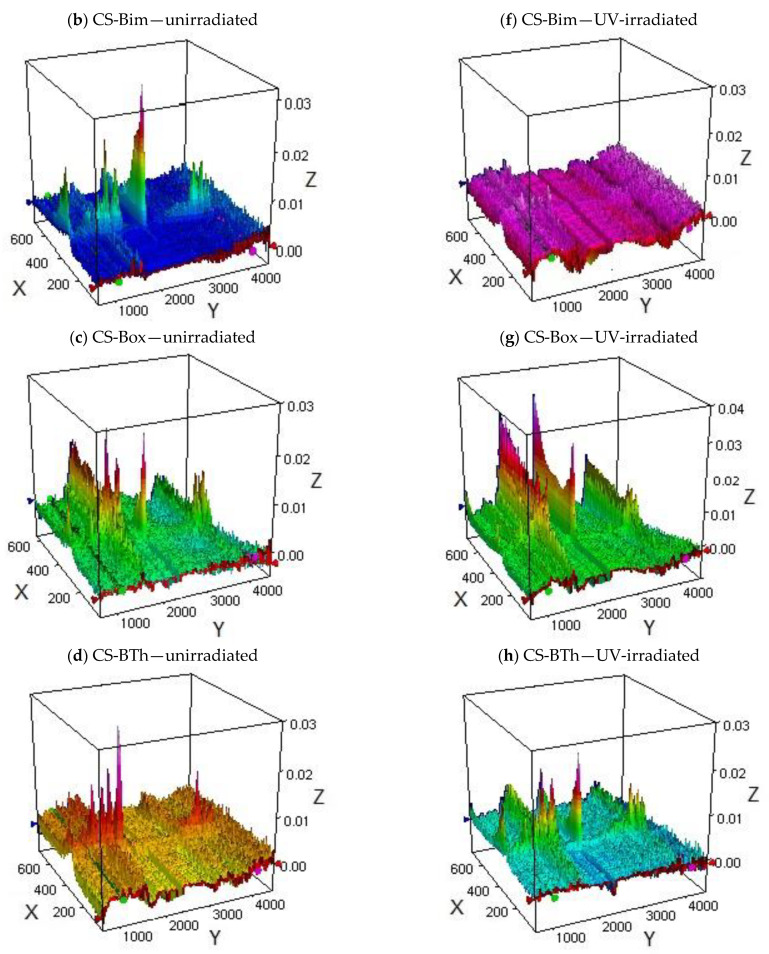
TGA-FTIR plots of CS, CS-BIm, CS-BOx, and CS-BTh samples: left panel—unirradiated samples (**a**,**c**,**e**,**g**); right panel—8 h UV irradiated samples (**b**,**d**,**f**,**h**). Axis description: X—temperature (°C), Y—wavenumber (cm^−1^), and Z—absorbance.

However, some differences are observed in the composition of volatile products released from the irradiated samples (Figure 6). Generally speaking, the most significant number of volatile products at temperatures above 250 °C was released from the starting (unexposed) chitosan (Figure 6a). The main bands are observed for unirradiated CS alone at the values (in cm^−1^) of 3520–3630 (OH), 2260–2400 (CO_2_), 1680–1840 (C=O, amide), 1390, 1280, 1174 (C-O), and 972 (N-H). Thus, the products are a mixture of water, carbon dioxide, ammonia, acetamide, and low-molecular carbonyl (ester)/ether derivatives.

This is in line with the results published by Corazzari et al. [43], who confirmed the release of H_2_O, CO, CO_2_, NH_3,_ and CH_3_COOH during the pyrolysis of chitosan using the TGA-FTIR and GC-MS methods. Only carbon monoxide and acetic acid were not detected in our experiment. In another study, apart from the above-mentioned compounds, acetamide was found, the amount of which depends on the degree of CS deacetylation [44]. In an earlier work by Zeng et al., heterocyclic aromatic compounds with nitrogen, mainly pyrazines, were also detected during an isothermal test at 553 K [45]. Such products were not found in this work.

In UV-irradiated CS, similar bands were observed, but they were much less intensive (Figure 6b). This allows us to conclude that the identified products partially evolved during the photolysis preceding the thermal decomposition.

The FTIR of the gaseous excretions from CS-BIm (Figure 6c), besides the substances typical for CS, also contain a significant number of unsaturated compounds (absorbing at 953 cm^−1^), but in this irradiated sample, their release is minimized (Figure 6d).

The exposure to UV radiation only resulted in a 2–6.5 °C increase in T_max_ in the CS alone, CS-BIm, and CS-Box samples, while in the CS-BTh sample, the T_max_ decreases by two degrees.

Further, the loss of mass during the thermal decomposition of the irradiated samples differs slightly from that in the non-irradiated ones. It can therefore be concluded that the samples of modified CS are resistant to heat and, like chitosan itself, decompose only above 250 °C.

The unexposed CS-BOx and CS-BTh samples (Figure 6e,g) show less CO_2,_ but the detached carbonyl product is dominant. Only in CS-BOx is a more volatile product after the exposure to UV observed (Figure 6f), which may indicate the catalytic effect of the photodegradation products trapped in the polymer matrix on the thermal destruction of this specimen. According to Bussiere’s reports [46,47], the released volatile products can accumulate on the surface of the samples and contribute to an increase in roughness and adhesion work. The authors emphasize that the observed changes are the effect of the surface crosslinking.

The order of the thermal stability of the irradiated chitosan derivatives (estimated from the temperatures at a maximum decomposition rate of IInd step—T_max_, Table 3) is:

CS-BOx > CS-BTh ≅ CS > CS-BIm

It practically does not differ from that for the non-irradiated samples.

## 4. Conclusions

Thermogravimetry coupled with an FTIR analysis of volatile decomposition products indicated that BIm is the most thermally stable sample, and BOx is the least thermally resistant sample among the three tested dyes used for CS modification. At the same time, the BIm sample released the most volatile products, containing heteroatoms (mainly oxygen and nitrogen) and unsaturated bonds.

Heterocyclic aromatic substituents only negligibly contribute to the heat resistance of the CS. The TGA analysis showed the resistance of chitosan derivatives up to ca. 250 °C. Based on the temperatures at the beginning and the maximum decomposition rate, it can be stated that the lowest thermal stability is exhibited by CS-BIm, which is related to its lower susceptibility to thermal crosslinking. The other two derivatives (CS-BOx and CS-BTh) also showed a lower mass at 600 °C (compared to CS alone), which is a carbonized crosslinked biopolymer residue.

The effect of UV exposure on thermal stability was also studied. CS-BOx shows the best resistance among all UV-exposed and unexposed CS derivatives.

The main volatile products of the thermal decomposition of modified CS are water, carbon dioxide, ammonia, and carbonyl/ether compounds. The number of these evaporated products decreases in the UV-irradiated samples.

The good thermal and photochemical stability of the dye-substituted chitosan is crucial from a practical point of view. Thanks to this, such materials intended, for example, for biomedical applications, can be sterilized with heat or UV radiation.

## Figures and Tables

**Table 1:** Parameters of heterocyclic compounds (BIm, BOx, and BTh) determined from TG, DTG, DSC, and Gram–Schmidt curves.

Sample	T_o_, °C	T_max_, °C	∆m, %	V_max_, %/min	Melting Point, °C	Gram–Schmidt Curve
BIm	320	362	64.2	8.0	196	two peaks of similar intensity at 393 and 488 °C;
BOx	244	279, 300	98.9	19.3	187	two peaks at 342 °C (main) and 549 °C (weak);
BTh	265	310	96.7	17.5	156, 174	lack of peaks, monotonic growth without extremum

**Table 2:** Thermal parameters of unirradiated (0 h) chitosan and modified chitosan samples determined from TGA/DSC analysis.

Sample 0 h	I Step			II Step					Total ∆m, %
	T_o_, °C	T_max_, °C	∆m, %	T_o_, °C	T_max_, °C	∆m, %	T_exo_, °C; ΔH, J/g	V_max_, %/min	
CS	80	121	7.7	254	277	54.8	276; 153	7.5	62.4
CS-BIm	75	94	5.3	254	273	51.7	280; 172	7.3	55.6
CS-BOx	73	103	7.9	265	288	53.0	291; 140	8.7	60.5
CS-BTh	75	110	5.7	259	284	52.6	288; 132	7.4	58.3

**Table 3:** Thermal parameters of 8 h UV irradiated chitosan and modified chitosan samples determined from TGA analysis.

Sample 8 h UV	I Step			II Step					Total ∆m, %
	T_o_, °C	T_max_, °C	∆m, %	T_o_, °C	T_max_, °C	∆m, %	T_exo_,°C; ΔH, J/g	V_max_, %/min	
CS	83	92	8.1	254	283	52.9	283; 140	6.5	60.7
CS-BIm	73	94	5.3	254	276	51.7	286; 285	7.5	57.1
CS-BOx	73	101	5.6	266	290	53.5	296; 402	8.2	59.2
CS-BTh	71	103	5.6	255	280	52.2	287; 290	7.1	58.0

## Supplementary Materials

The following supporting information can be downloaded at: https://www.mdpi.com/article/10.3390/ma15103667/s1, Figure S1: Photos of thin films of studied samples: chitosan (a), CS-BIm (b), CS-BOx (c), CS-BTH (d).
