# Supplementary material for: Thermal Stability of Fluorescent Chitosan Modified with Heterocyclic Aromatic Dyes
Source: Materials (Basel). 2022 May 20;15(10):3667. doi: 10.3390/ma15103667 (PMC9147818; doi:10.3390/ma15103667)
Supplement: Supplementary file 1 [file materials-15-03667-s001.zip › materials-1701649-supplementary.pdf]

Supplementary Materials

Thermal stability of fluorescent chitosan modified with heterocyclic aromatic dyes

Dagmara Bajer and Halina Kaczmarek

Faculty of Chemistry, Nicolaus Copernicus University in Toruń,
Gagarina 7, 87-100 Toruń, Poland

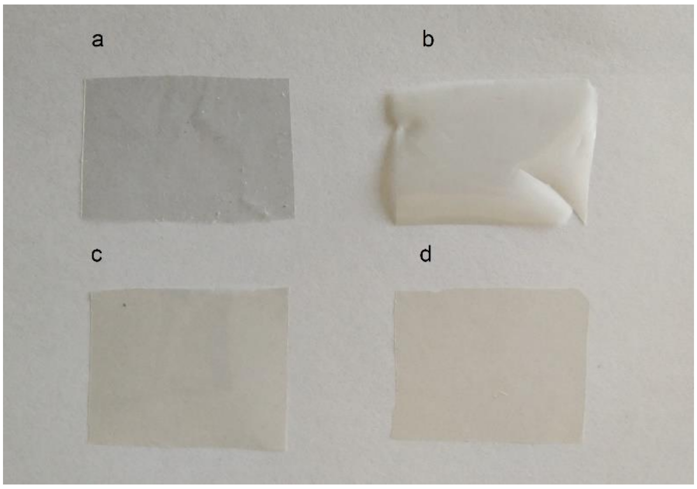

Figure S1. Photos of thin films of studied samples: chitosan (a), CS-BIm (b); CS-BOx (c), CS-BTh (d).
